# Novel role of a triglyceride-synthesizing enzyme: DGAT1 at the crossroad between triglyceride and cholesterol metabolism

**DOI:** 10.1016/j.bbalip.2016.06.014

**Published:** 2016-06-23

**Authors:** Vinay Sachdev, Christina Leopold, Raimund Bauer, Jay V. Patankar, Jahangir Iqbal, Sascha Obrowsky, Renze Boverhof, Marcela Doktorova, Bernhard Scheicher, Madeleine Goeritzer, Dagmar Kolb, Andrew V. Turnbull, Andreas Zimmer, Gerald Hoefler, M. Mahmood Hussain, Albert K. Groen, Dagmar Kratky

**Affiliations:** a Institute of Molecular Biology and Biochemistry, Medical University of Graz, 8010 Graz, Austria; b Center of Experimental Medicine, University Medical Center Hamburg-Eppendorf, 20246 Hamburg, Germany; c Centre for Molecular Medicine and Therapeutics, University of British Columbia, Vancouver, BC V5Z 4H4, Canada; d Department of Cell Biology, SUNY Downstate Medical Center, 11203 New York, United States; e Departments of Pediatrics, Center for Liver, Digestive, and Metabolic Diseases, University of Groningen, University Medical Center Groningen, 9713 Groningen, The Netherlands; f Institute of Pharmaceutical Sciences, University of Graz, 8010 Graz, Austria; g Institute of Cell Biology, Histology, and Embryology, Medical University of Graz, 8010 Graz, Austria; h AstraZeneca R&D, 431 50 Moelndal, Sweden; i Institute of Pathology, Medical University of Graz, 8010 Graz, Austria

**Keywords:** Cholesterol absorption, Chylomicron size, DGAT1 inhibition, Intestinal DGAT1 deficiency, Trans-intestinal cholesterol excretion

## Abstract

Acyl-CoA:diacylglycerol acyltransferase 1 (DGAT1) is a key enzyme in triacylglycerol (TG) biosynthesis. Here we show that genetic deficiency and pharmacological inhibition of DGAT1 in mice alters cholesterol metabolism. Cholesterol absorption, as assessed by acute cholesterol uptake, was significantly decreased in the small intestine and liver upon DGAT1 deficiency/inhibition. Ablation of DGAT1 in the intestine (I-DGAT1^−/−^) alone is sufficient to cause these effects. Consequences of I-DGAT1 deficiency phenocopy findings in whole-body DGAT1^−/−^ and DGAT1 inhibitor-treated mice. We show that deficiency/inhibition of DGAT1 affects cholesterol metabolism via reduced chylomicron size and increased trans-intestinal cholesterol excretion. These effects are independent of cholesterol uptake at the apical surface of enterocytes but mediated through altered dietary fatty acid metabolism. Our findings provide insight into a novel role of DGAT1 and identify a pathway by which intestinal DGAT1 deficiency affects whole-body cholesterol homeostasis in mice. Targeting intestinal DGAT1 may represent a novel approach for treating hypercholesterolemia.

## 1 Introduction

Acyl-CoA:diacylglycerol acyltransferase (DGAT)1 and 2 catalyze the final and rate-limiting step of triacylglycerol (TG) biosynthesis mediated mainly through the 2-monoacylglycerol and glycerol-phosphate pathways [1]. The two DGAT enzymes DGAT1 and DGAT2 are encoded by two different genes belonging to distinct acyl-transferase families, which lack significant sequence homology [2]. Highest expression levels of both genes are found in tissues active in TG synthesis, such as adipose tissue, small intestine, liver, and mammary gland [3]. In the intestine, DGAT1 is predominantly involved in the biosynthesis of TG from dietary fatty acids (FA) [4]. In the postprandial state, esterified lipids generated by intestinal DGAT1 are incorporated into chylomicron particles, secreted into the lymphatic system, and subsequently released into the peripheral circulation via the thoracic duct [5]. DGAT1 knockout (DGAT1^−/−^) mice are viable, resistant to diet-induced obesity and fatty liver disease, exhibit increased energy expenditure, and normal insulin and leptin sensitivity [3,6]. Mice that express DGAT1 exclusively in the small intestine are, however, susceptible to high-fat diet-induced hepatic steatosis and obesity [7]. Loss of DGAT1 specifically in the intestine leads to reduced postprandial TG and retinyl ester concentrations due to reduced chylomicron secretion [8]. These observations implicate intestinal DGAT1 as a potential target to reduce postprandial lipemia by altering chylomicron biosynthesis [9].

Pharmacological DGAT1 inhibition studies in animal models have consistently demonstrated reduction in diet-induced weight gain and postprandial chylomicron secretion [10,11]. Pharmacological inhibition of DGAT1 was also shown to reduce plasma total cholesterol (TC) concentrations in mice [12]. The utility of DGAT1 inhibitors has recently been strengthened by studies in patients suffering from familial chylomicronemia [13]. Thus, DGAT1 is a potential target to improve metabolic parameters in hyperlipidemic states. Intestinal DGAT1 has been speculated to exert these effects by increasing the secretion of gut hormones, such as glucagon-like peptide-1 and peptide YY [14]. However, endocrine changes alone are unable to explain the observed postprandial phenotype upon pharmacological DGAT1 inhibition and thus the precise mechanisms regulating DGAT1 inhibitory effects still remain elusive [8].

We have previously reported that DGAT1 deficiency in apolipoprotein E^−/−^ mice has an athero-protective role. These beneficial effects included lower plasma cholesterol concentrations and reduced intestinal cholesterol absorption [15]. Given its predominant role in TG biosynthesis, the profound reduction in cholesterol uptake and absorption is puzzling. Our results indicate that inhibition or ablation of DGAT1 in enterocytes is sufficient to reduce the incorporation of cholesterol esters (CE) into chylomicrons. We speculate that the reduced chylomicron size further facilitates the trans-intestinal excretion of cholesterol (TICE) upon DGAT1 inhibition conferring athero-protection.

## 2 Materials and methods

### 2.1 Animals and diets

Age-matched male mice (8–12 weeks of age) were used for all experiments unless indicated. Mice had ad libitum access to water and food and were maintained under a 12-h light/12-h dark cycle in a temperature-controlled environment. I-DGAT1^−/−^ [8] and DGAT1^−/−^ [16] mice were generously provided by Dr. Robert Farese, Jr. (Harvard T.H. Chan School of Public Health, Boston, MA). LDLR^−/−^ mice (purchased from Jackson Laboratory, Bar Harbor, ME) were crossed with DGAT1^−/−^ mice to generate DGAT1^−/−^ LDLR^−/−^ mice. All mice were fed either regular chow diet (11.9% caloric intake from fat; Altromin 1324, Lage, Germany) or challenged with a high-fat/high-cholesterol diet (HF/HCD; 30% caloric intake from fat plus 1% cholesterol; Ssniff, Soest, Germany) starting at the age of 8 weeks. The fatty acid composition of the HF/HCD is delineated in supplementary Table S1.

Experiments to determine cholesterol fluxes upon chronic pharmacological DGAT1 inhibition were performed in compliance with national laws and were approved by the respective Ethical Committees for Animal Experiments, University of Groningen, The Netherlands. For all other studies, animal experiments were approved by the Austrian Federal Ministry of Science, Research, and Economy, Vienna, Austria, in accordance with the Council of Europe Conventions.

### 2.2 Blood biochemical analyses

Blood was collected from 4 h (6 a.m. until 10 a.m.) fasted mice and plasma was prepared within 20 min. TG, TC, and free cholesterol (FC) concentrations were measured enzymatically according to manufacturer’s instructions (DiaSys, Holzheim, Germany). Lipoprotein fractions were separated from 200 µl pooled plasma from each group using fast protein liquid chromatography (Pharmacia P-500) equipped with a Superose 6 column (Amersham Biosciences, Piscataway, NJ). TC concentrations in the isolated fractions were measured enzymatically.

### 2.3 Acute pharmacological DGAT1 inhibition

The DGAT1 inhibitor used in this study has been described elsewhere as compound 2 [11]. Dosage for the inhibitor treatment was chosen based on a previous report [8]. Treatments were initiated one day before start of the experiment at 8 a.m. after a 2 h fasting period and were repeated every 24 h during the course of the experiment. Mice had access to food 2 h post treatment. Wild-type mice on a C57BL/6 background were orally dosed with either vehicle composed of 0.5% (w/v) of hydroxypropylmethylcellulose (Sigma-Aldrich, St. Louis, MO) in 0.1% (v/v) Tween 80 or DGAT1 inhibitor (5 mg/kg body weight) dissolved in vehicle. On the day of cholesterol absorption experiments (described below) mice were gavaged with corn oil containing radiotracers 2 h post DGAT1 inhibitor treatment.

### 2.4 Short-term cholesterol absorption

Cholesterol absorption studies were performed as described previously [15]. Briefly, chow diet-fed mice were fasted for 4 h and gavaged with 200 µl corn oil containing 2 µCi [^3^H]cholesterol (ARC Inc., St Louis, MO) and 200 µg cholesterol. After 4 h, plasma, liver, and three parts of the small intestine were isolated. Duodenum, jejunum, and ileum were rinsed with PBS to remove luminal contents. The tissues were dissolved in 1 M NaOH at 65 °C overnight for protein quantitation and analyzed by liquid scintillation counting.

### 2.5 Fractional cholesterol absorption

Fractional cholesterol absorption was measured by the fecal dualisotope ratio method as described [15]. Chow diet-fed mice were fasted for 4 h and gavaged with a single dose of 200 µl corn oil containing 0.2 µCi [^3^H]sitostanol (ARC Inc., St. Louis, MO) and 0.1 µCi [^14^C]cholesterol (ARC Inc., St. Louis, MO). Feces were collected for 72 h and lipids were extracted using the Folch extraction method. Radioactivity in fecal samples was measured by liquid scintillation counting. Fractional cholesterol absorption was calculated using the following formula: % absorption = (dose [^14^C]:[^3^H] – fecal [^14^C]:[^3^H])/ dose [^14^C]:[^3^H] × 100.

### 2.6 Uptake and secretion of cholesterol by primary enterocytes

Primary enterocytes were isolated from overnight fasted C57BL/6 mice, suspended in 4 ml of DMEM, and incubated at 37 °C as described [17]. DMEM was supplemented with either vehicle (DMSO) or DGAT1 inhibitor (EC_50_ = 0.03 µm, dissolved in DMSO) and 1 µCi/ml [-^3^H]cholesterol suspended in mixed lipid micelles (0.14 mM sodium cholate, 0.15 mM sodium deoxycholate, 0.17 mM phosphatidylcholine, 0.19 mM monooleoylglycerol). Cholesterol uptake was determined at indicated time points, after which enterocytes were washed, cellular lipids were extracted, and radioactivity was measured by liquid scintillation counting. For determination of cholesterol secretion, enterocytes were labeled for 1 h and chased with medium containing secretion micelles (1.4 mM oleic acid, 0.14 mM sodium cholate, 0.15 mM sodium deoxycholate, 0.17 mM phosphatidylcholine, 0.19 mM mono-oleoylglycerol) in the absence and presence of DGAT1 inhibitor. After 2 h of chase, enterocytes were centrifuged, lipids were extracted from the supernatant and separated by thin layer chromatography to quantify enterocyte [^3^H]cholesterol secretion into FC and cholesteryl ester (CE) fractions [17].

### 2.7 Chylomicron secretion

Chylomicron secretion rate using [9, 10-^3^H(N)]triolein (Perkin Elmer, Boston, MA) and [^14^C]cholesterol (ARC Inc., St. Louis, MO) was assessed as previously described [8] with minor modifications. Briefly, mice fed HF/HCD for 12 weeks were fasted for 4 h starting at 6 a.m. Thereafter, they were intraperitoneally injected with Poloxamer-407 (P-407; 1 g/kg body weight, Sigma-Aldrich, St. Louis, MO) in PBS and gavaged with 200 µl corn oil containing 1 µCi [^3^H]triolein, 0.5 µCi [-^14^C]cholesterol, and 200 µg cholesterol. Prior to P-407 injection and post 1, 2, 3, and 4 h corn oil gavage, blood samples were collected and radioactivity was measured in the plasma. Four hours post gavage, mice were anaesthetized and lymph was surgically removed as described [18] with modifications. Briefly, anaesthetized mice were aseptically prepared for surgery and the abdomen was opened through a left subcoastal incision. A self-retaining retractor was placed to cranially mobilize spleen, liver, and stomach to expose the cisterna chyli and the thoracic duct-containing postprandial milky lymph. Under a dissecting microscope, lymph was carefully collected using glass microcapillary tubes. Radioactivity in a pool of 100 µl lymph was measured by liquid scintillation counting.

### 2.8 Chylomicron size measurement

Mice fed HF/HCD for 12 weeks were fasted for 4 h starting at 6 a.m., intraperitoneally injected with P-407, and gavaged with 200 µl corn oil. Ninety minutes post gavage, blood and lymph were isolated as described above. Plasma chylomicrons were separated by densitygradient centrifugation as described [19]. Briefly, 0.4 ml of plasma was mixed with 0.9 ml PBS (containing 2 mM benzamidine) and 0.7 g KBr (4 M final concentration, density 1.3 g/ml). The mixture was placed into 5.5 ml Quick-Seal centrifugation tubes (Beckman Coulter, Brea, CA), carefully overlayed with 0.9% NaCl, and centrifuged for 45 min at 416,000g (65,000 rev/min; VTi 65.2 rotor). Fractions were collected from the bottom of the tubes and the densities were determined. The top fraction contained chylomicrons. We processed the chylomicron-containing fraction for particle size analysis [19] by dynamic light scattering using a Zetasizer Nano ZS (Malvern Instruments Ltd., Malvern, UK) at 25 °C according to manufacturer’s instructions. Particle size measurements were performed in triplicate and averaged. Data represent averages from 4 independent experiments and are indicated as mean hydrodynamic diameter in nanometer scale (Z-Average d_nm_).

### 2.9 Hematoxylin and eosin staining

Jejuna of overnight fasted HF/HCD-fed DGAT1^fl/fl^ and I-DGAT1^−/−^ mice were fixed in 4% neutral-buffered formaldehyde for 24 h and embedded in paraffin. Sections (5 µm) were deparaffinized and subjected to hematoxylin and eosin staining.

### 2.10 Freeze-fracture transmission electron microscopy

Five microliters of postprandial lymph and plasma chylomicrons from HF/HCD-fed mice were subjected to freeze-fracture transmission electron microscopy as described previously [20]. Images were taken using a FEI Tecnai G2 20 transmission electron microscope (FEI, Eindhoven, The Netherlands) with a Gatan ultrascan 1000 CCD camera. Acceleration voltage was 120 kV. Particle size distribution of 100–200 particles from 18 electron micrographs was estimated using Image J software (ImageJ 1.43U, NIH, USA).

### 2.11 Quantification of fecal neutral sterol loss, bile acid and fatty acid concentrations

Feces were collected for 72 h from individually housed mice. Fifty micrograms of dried feces were used for determination of fecal neutral sterol loss (NSL), bile acid (BA) concentrations, and FA composition by gas chromatography as described recently [21,22].

### 2.12 Measurement of TICE

C57BL/6 mice were purchased from Harlan (Horst, The Netherlands) and fed high-fat diet (HFD; 60% caloric intake from fat; Research Diet Services, Wijk Bij Duurstede, The Netherlands) supplemented with either vehicle or DGAT1 inhibitor (5 mg/kg diet) for 4 weeks. The fatty acid composition of the HFD is delineated in Table S1. Cholesterol flux experiments to measure TICE were performed as described [23].Briefly, mice were orally gavaged at day 0 with 1.5 µmol cholesterol-D5 (Medical Isotopes, Inc., Pelham, NH, USA) dissolved in MCT oil (Pharmacy UMCG, Groningen, The Netherlands) and were intravenously injected with 0.7 µmol cholesterol-D7 (Cambridge Isotope Laboratories, Inc., Andover, MA, USA) dissolved in Intralipid (20%, Fresenius Kabi, Den Bosch, The Netherlands). Blood spots were collected from the tail daily for 10 days. On day 10, mice were anaesthetized and hepatic bile was surgically collected for 20 min from the common bile duct via the gallbladder as described previously [23]. Tissues were excised and feces were collected from individual mice for 72 h prior to termination. Fecal NSL, BA, and FA concentrations were measured as described above.

### 2.13 Statistics

Statistical analyses were performed using GraphPad Prism 5.0 software (San Diego, CA). Data are presented as mean ± SEM. Comparisons between 2 groups were measured using unpaired 2-tailed Student’s *t*-test. Comparisons of multiple groups were analyzed by 2-way ANOVA followed by Bonferroni pos*t*-tests. Significance levels were set at *p* < 0.05 (*), *p* ≤ 0.01 (**), and *p* ≤ 0.001 (***).

## 3 Results

### 3.1 Intestinal DGAT1 deficiency reduces cholesterol absorption to levels that are comparable to global DGAT1 deficiency and pharmacological DGAT1 inhibition

**Fig. 1:**
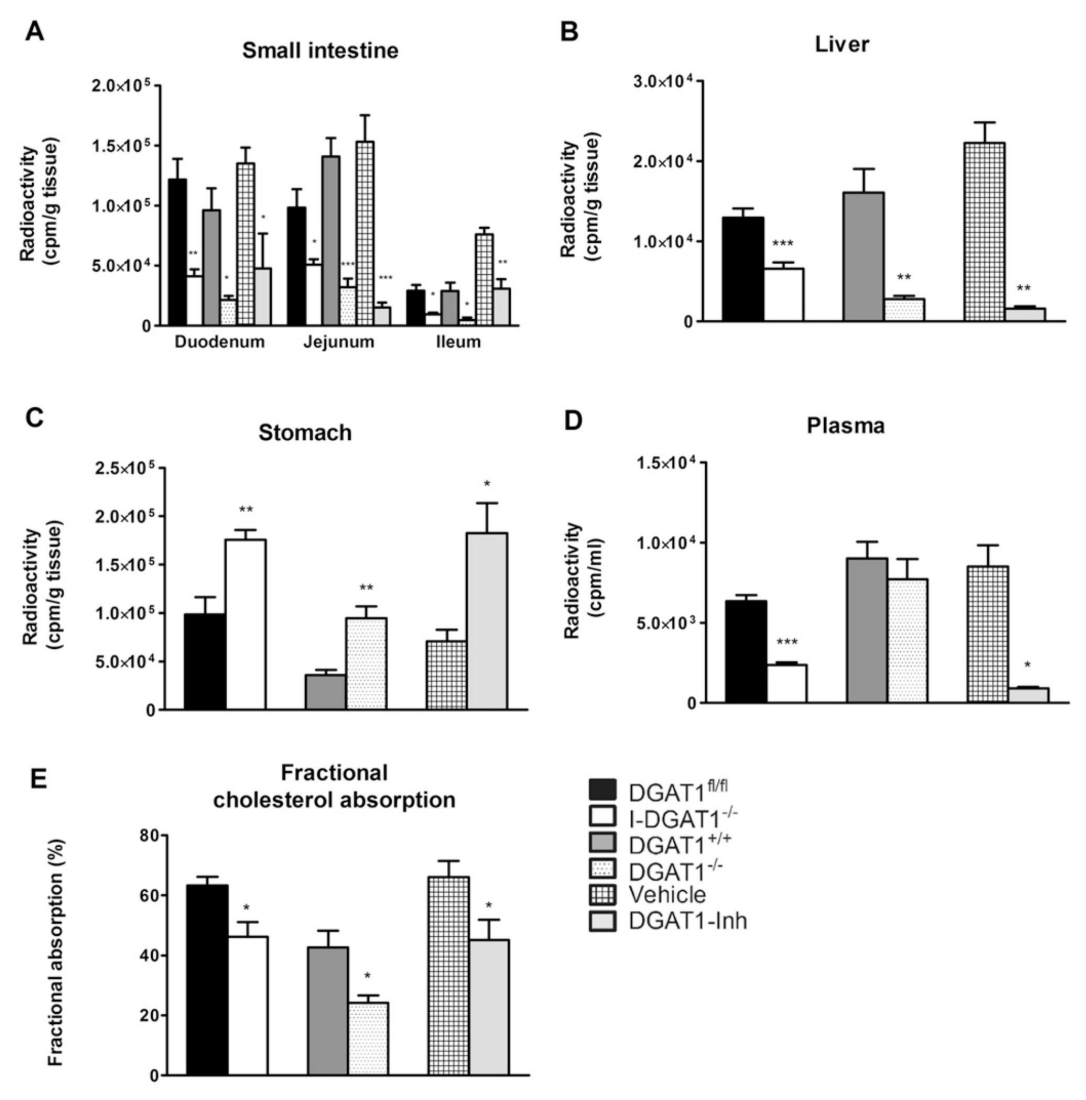
Intestinal DGAT1 deficiency reduces cholesterol absorption comparable to global DGAT1 deficiency and pharmacological DGAT1 inhibition. (A–D) Mice (*n* = 4–7) were gavaged with 200 µl corn oil containing 2 µCi [^3^H]cholesterol and 200 µg cholesterol. Radioactivity was measured 4 h post-gavage in (A) duodenum, jejunum, ileum, (B) liver, (C) stomach, and (D) plasma by liquid scintillation counting. (E) Fractional cholesterol absorption was determined using the fecal dual-isotope method (*n* = 5–10). Values represent mean + SEM. Student’s *t*-test was used to compare grouped controls. *p < 0.05, **p < 0.01, ***p < 0.001.

To analyze cholesterol metabolism, we first investigated short-term and fractional cholesterol absorption between I-DGAT1^−/−^, DGAT1^−/−^, and DGAT1-Inh mice and their respective controls. Absence or inhibition of DGAT1 resulted in significantly decreased acute cholesterol absorption as indicated by reduced radioactivity in all parts of the small intestine by >50% and in liver (50%, 82%, and 92%, respectively) of I-DGAT1^−/−^, DGAT1^−/−^, and DGAT1 inhibitor-treated (DGAT1-Inh) mice (Fig. 1A, B).

The radiotracer was retained considerably in the stomachs of I-DGAT1^−/−^, DGAT1^−/−^, and DGAT1-Inh mice compared to their respective controls (1.7-fold, 2.6-fold, and 2.6-fold, respectively) (Fig. 1C). In addition, we observed reduced radioactivity in the plasma of I-DGAT1^−/−^ and DGAT1-Inh mice (73% and 90%, respectively) (Fig. 1D). We then measured fractional cholesterol absorption in these mice by using the fecal dual-isotope method, in which feces were collected for 72 h after a single intragastric bolus of radiolabeled cholesterol and sitosterol. As shown in Fig. 1E, fractional cholesterol absorption was reduced in I-DGAT1^−/−^, DGAT1^−/−^, and DGAT1-Inh mice by 27%, 43%, and 32%, respectively, compared to their respective controls.

### 3.2 Intestinal DGAT1 deficiency reduces plasma cholesterol levels in HF/HCD-fed mice

**Fig. 2:**
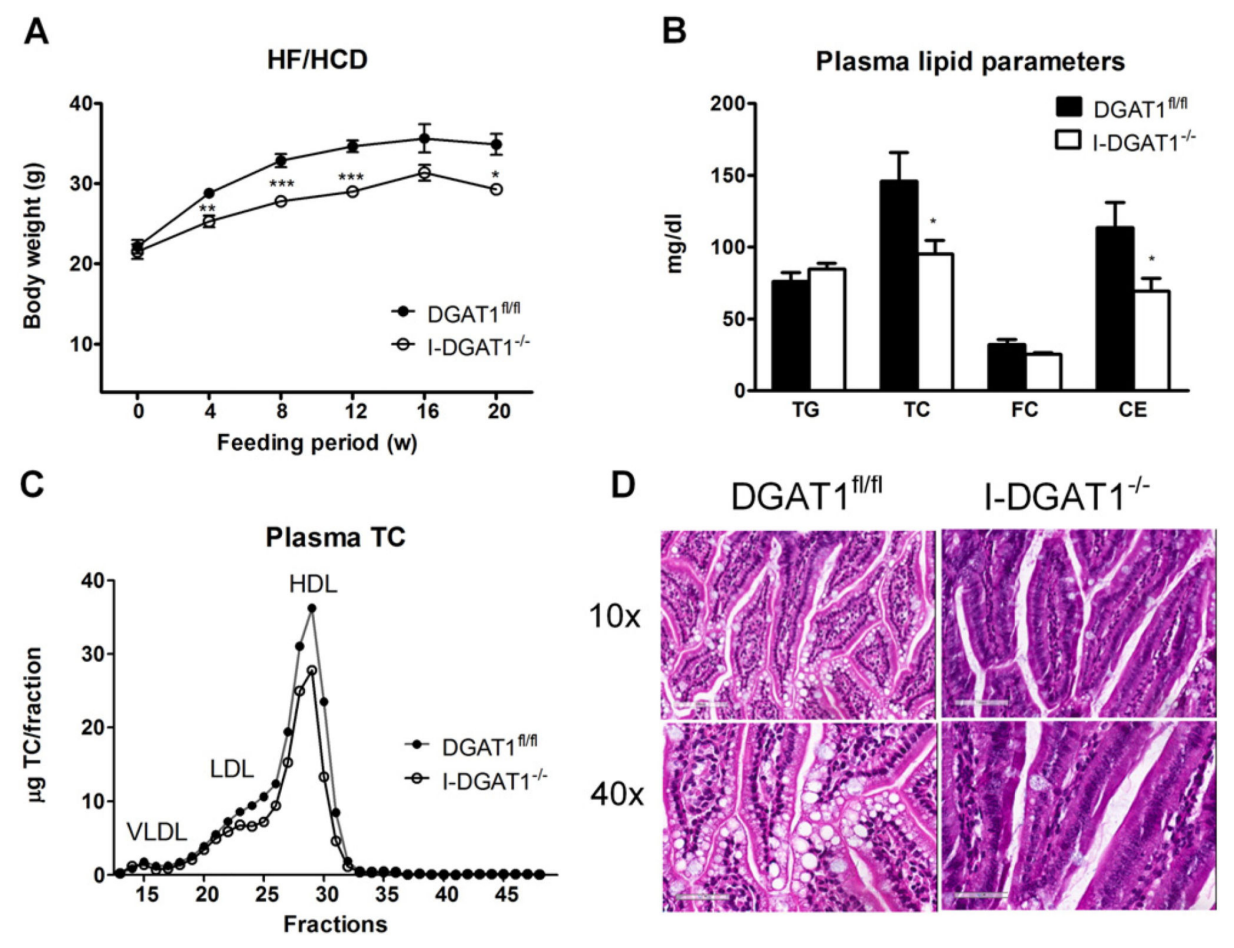
Intestinal DGAT1 deficiency reduces plasma cholesterol concentrations. DGAT1^fl/fl^ and I-DGAT1^−/−^ mice were fed HF/HCD for 20 weeks. (A) Body weights (*n* = 17–19 until week 12, *n* = 5–6 until week 20). (B) Plasma TG, TC, and FC concentrations were measured spectrophotometrically. CE concentrations were determined as subtraction of FC from TC (*n* = 5–6). (C) Lipoprotein profile of TC after separation by fast performance liquid chromatography of pooled plasma (*n* = 5–6). (D) Hematoxylin and eosin staining of duodenal sections. Top panel: magnification, 10×; scale bar, 200 µm. Bottom panel: magnification, 40×; scale bar, 50 µm. Values represent mean ± SEM. A: 2-way ANOVA followed by Bonferroni post-test; B: Student’s *t*-test. *p < 0.05, **p < 0.01, ***p < 0.001.

Previous reports have described reduced body-weight gain and improved metabolic profile upon DGAT1 deficiency when mice were fed with increased dietary fat [3,4]. We hypothesized that challenging global and intestinal DGAT1-deficient mice with increased fat and cholesterol will mimic these observations. Thus, we investigated the impact of intestinal DGAT1 deficiency on cholesterol metabolism in HF/HCD-fed mice over a period of 20 weeks. Body-weight gain was reduced in I-DGAT1^−/−^ mice compared to controls starting 4 weeks after initiation of the diet and sustained until the end of the feeding period (Fig. 2A). Moreover, upon 4 h of fasting, I-DGAT1^−/−^ mice exhibited comparable plasma TG but reduced TC and CE concentrations (35% and 39%, respectively) compared to DGAT1^fl/fl^ mice (Fig. 2B). Plasma lipoprotein profiles after separation by fast protein liquid chromatography revealed decreased LDL and HDL cholesterol levels in I-DGAT1^−/−^ mice (Fig. 2C). To investigate whether intestinal DGAT1 deficiency had any impact on small intestinal morphology we stained intestinal sections with hematoxylin and eosin. As shown in Fig. 2D, we observed intact villi morphology in I-DGAT1^−/−^ mice with reduced lipid accumulation. We observed comparable results in whole-body DGAT1^−/−^ mice and DGAT1-LDLR^−/−^ mice (Supplementary Fig. 1A–C and Supplementary Fig. 2A, B).

### 3.3 DGAT1 inhibition reduces CE secretion from enterocytes

**Fig. 3:**
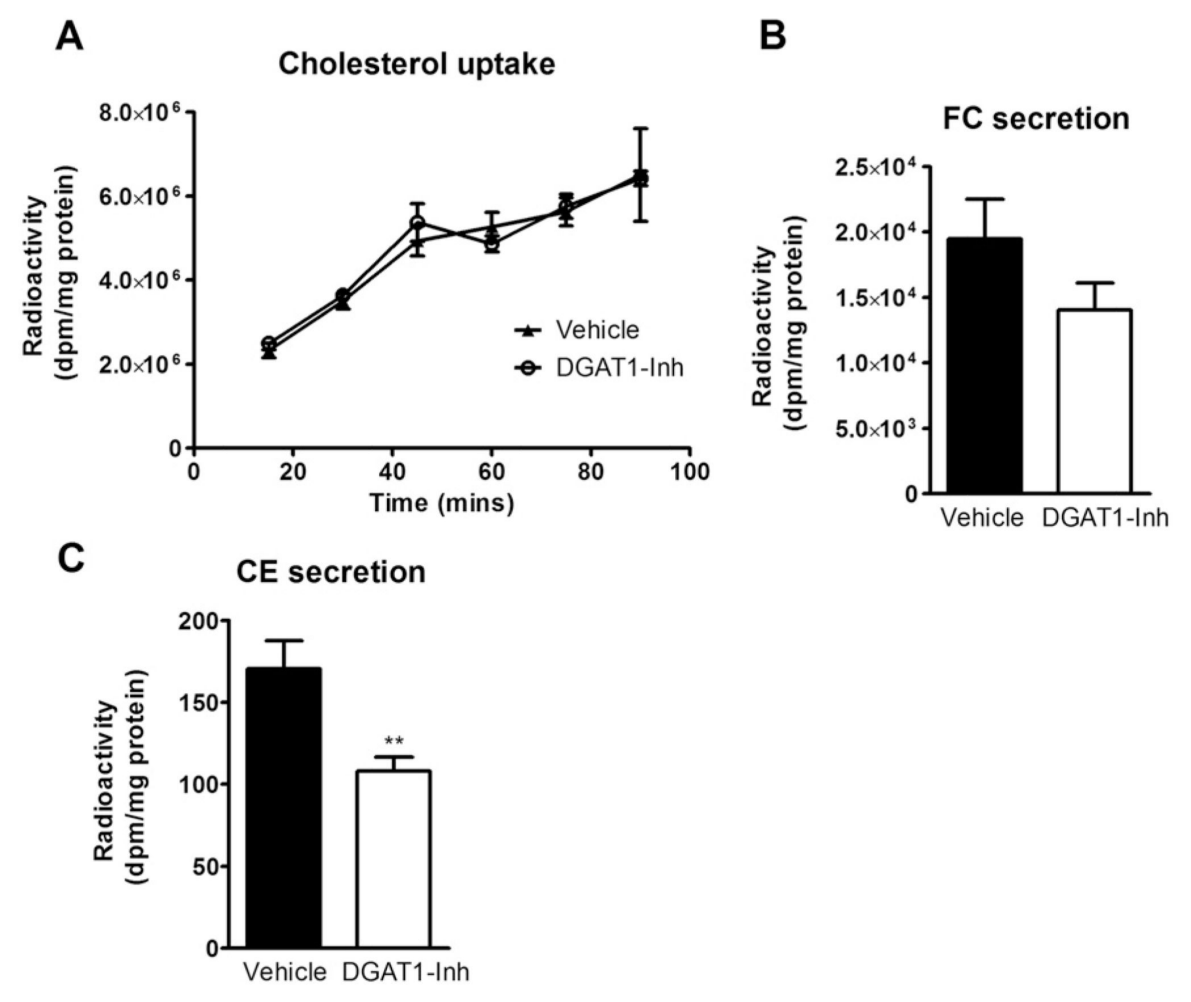
DGAT1 inhibition reduces enterocyte cholesteryl ester secretion. Enterocytes were isolated from overnight fasted C57BL/6 mice, treated with either vehicle or DGAT1-Inh (EC_50_ = 0.03 µM), and radiolabeled with 1 µCi [^3^H]cholesterol/ml. (A) Enterocyte [^3^H]cholesterol uptake was measured at indicated time points (*n* = 3). (B, C) One hour after [^3^H]cholesterol uptake, enterocytes were washed and chased with media containing 1.4 mM oleic acid micelles for 2 h. Lipids were isolated, subjected to TLC, and radioactivity was counted in (B) FC and (C) CE fractions. A: 2-way ANOVA followed by Bonferroni post-test; B, C: Student’s *t*-test. **p < 0.01.

We next investigated whether cholesterol retention in the stomach is the primary cause of reduced cholesterol absorption or whether DGAT1 deficiency in enterocytes alters cholesterol metabolism independent of gastric emptying. Thus, we performed ex vivo enterocyte cholesterol uptake and secretion studies upon DGAT1 inhibition in enterocytes isolated from wild-type mice. We observed unaltered cholesterol uptake in DGAT1-Inh enterocytes compared to vehicle-treated enterocytes (Fig. 3A). Secretion of both FC and CE was reduced in DGAT1-Inh enterocytes by 27% and 32%, respectively, compared to vehicle-treated enterocytes (Fig. 3B, C). These results indicate that DGAT1 activity in enterocytes directly regulates cholesterol uptake and absorption.

### 3.4 Intestinal DGAT1 deficiency alters chylomicron size and lymph lipid content

**Fig. 4:**
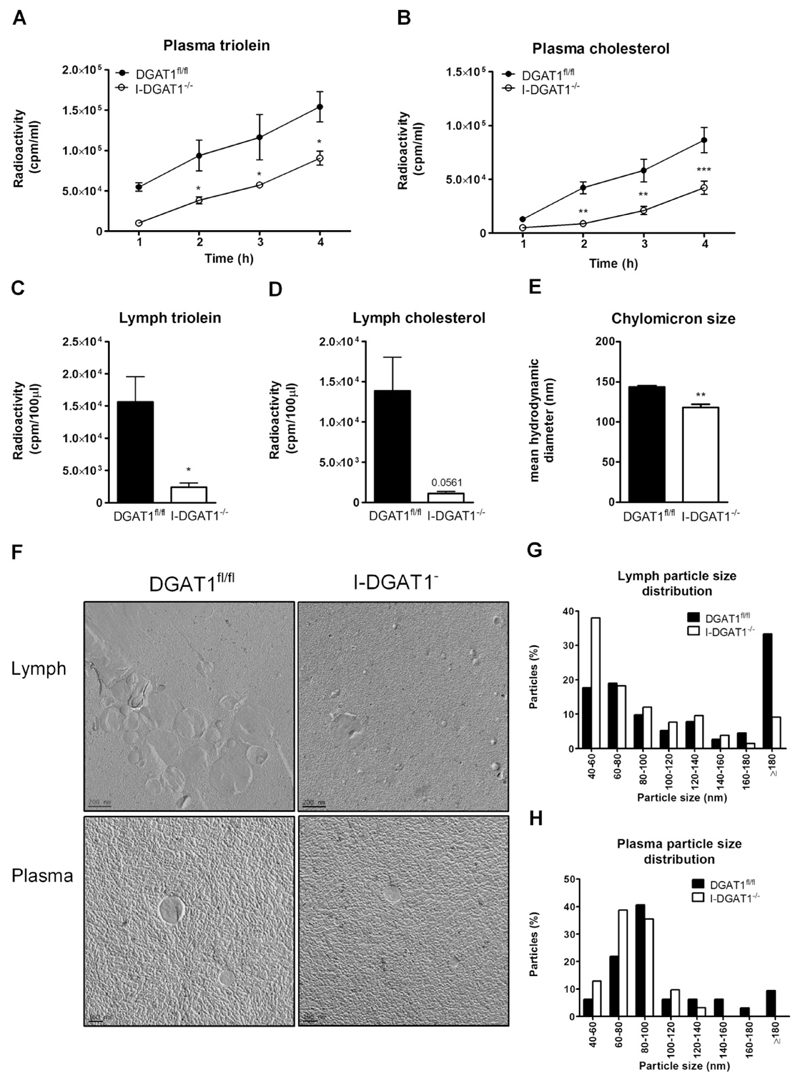
Intestinal DGAT1 deficiency alters chylomicron size and lymph lipid composition. (A–D) DGAT1^fl/fl^ and I-DGAT1^−/−^ mice (*n* = 4) fed HF/HCD were fasted for 4 h, injected intraperitoneally with poloxamer-407 (1 g/kg body weight, in PBS), and gavaged with 200 µl corn oil containing 1 µCi [^3^H]triolein, 0.5 µCi [^14^C]cholesterol, and 200 µg cholesterol. (A, C) [^3^H]triolein-derived counts in (A) plasma and (C) lymph and (B, D) [^14^C]cholesterol-derived counts in (B) plasma and (D) lymph were determined by liquid scintillation counting. (E–H) Plasma and lymph was collected 90 min post-gavage of 200 µl corn oil. (E) Postprandial plasma chylomicron size was measured by Zetasizer nano. (F) Electron micrographs of isolated lymph (top panel) and plasma chylomicrons (bottom panel). Distribution of particles size of (G) lymph and (H) plasma chylomicrons from 18 electron micrographs was analyzed by image J software. Values represent mean ± SEM. A, B: 2-way ANOVA followed by Bonferroni post-test; C-E: Student’s *t*-test. *p < 0.05, **p < 0.01, ***p < 0.001.

Newly synthesized post-prandial chylomicrons from the intestine are first transported to the lymph before being released into systemic circulation [5]. Thus, we administered mice with radiolabeled triolein and cholesterol, chased post-prandial plasma, and collected lymph 4 h post-administration of the radiotracers. Radioactive counts from lymph and plasma may provide a rough estimate of administered TG and CE incorporated into the newly synthesized chylomicrons. In fact, secretion of [^3^H]triolein- (Fig. 4A) and [^14^C]cholesterol-derived counts (Fig. 4B) were drastically reduced in plasma of I-DGAT1^−/−^ compared to DGAT1^fl/fl^ mice. In addition, [^3^H]triolein- and [^14^C]cholesterol-derived counts in the lymph were decreased by 85% and 92%, respectively (Fig. 4C, D). We then isolated plasma chylomicrons and lymph from P-407-treated mice after 90 min of fat challenge and subjected them to dynamic light scattering (DLS) and electron microscopy to estimate their size. Plasma chylomicrons of I-DGAT1^−/−^ mice were significantly (20%) smaller as measured by DLS (Fig. 4E). The polydispersity index for all measured samples was between 0.1 and 0.2 indicating narrow size distribution of particles. Visualization of chylomicrons in lymph (Fig. 4F, top panel) and plasma (Fig. 4F, bottom panel) by electron microscopy and their quantification confirmed smaller particle size in I-DGAT1^−/−^ compared to DGAT1^fl/fl^ mice (Fig. 4G, H). We observed similar reductions in chylomicron size in DGAT1^−/−^ mice (Supplementary Fig. S3).

### 3.5 Intestinal DGAT1 deficiency leads to increased fecal NSL and FA excretion in HF/HCD-fed mice

**Fig. 5:**
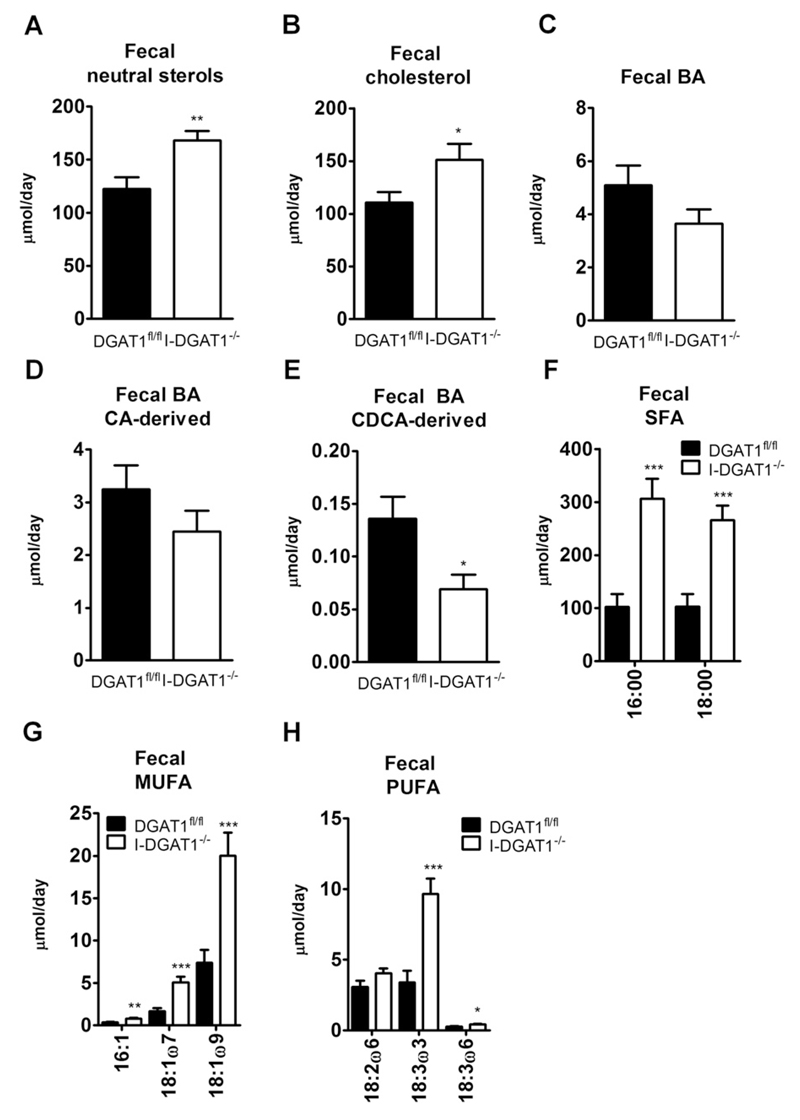
Intestinal DGAT1 deficiency leads to increased fecal neutral sterol and fatty acid excretion in HF/HCD-fed mice. Feces of HF/HCD-fed mice (*n* = 10–12) were collected for 72 h. Neutral sterols, bile acids (BA), and fatty acids (FA) were measured by gas chromatography. (A) Total fecal neutral sterols, (B) cholesterol, (C) total BA, (D) cholic acid (CA)-derived BA, and (E) chenodeoxycholic acid (CDCA)-derived BA, (F) saturated FA (SFA), (G) mono-unsaturated FA (MUFA), and (H) poly-unsaturated FA (PUFA) are indicated. Values represent mean + SEM. *p < 0.05, **p < 0.01, ***p < 0.001, Student’s t-test.

Since reduced cholesterol absorption in mice has been shown to increase fecal NSL [23], we measured fecal NSL and FA excretion in feces collected from DGAT1^fl/fl^ and I-DGAT1^−/−^ mice fed HF/HCD for 12 weeks. Total fecal NSL was 1.4-fold higher in I-DGAT1^−/−^ mice (Fig. 5A). In accordance, fecal cholesterol was significantly increased in I-DGAT1^−/−^ mice (Fig. 5B). Fecal total BA (Fig. 5C) and cholic acid (CA)-derived BA (Fig. 5D) concentrations were comparable in both genotypes. Fecal chenodeoxycholic acid (CDCA)-derived BA levels were reduced in I-DGAT1^−/−^ mice (Fig. 5E). Accordingly, we observed no changes in hepatic Cyp7a1 mRNA expression in I-DGAT1^−/−^ compared to DGAT1^fl/fl^ mice (supplementary Fig. S4). Fecal excretion of saturated FA (SFA), mono-unsaturated FA (MUFA), and poly-unsaturated FA (PUFA) was markedly increased in I-DGAT1^−/−^ mice (Fig. 5F-H). mRNA and protein expression levels ofseveral candidate genes, however, were unaltered in duodena of I-DGAT1^−/−^ mice (Supplementary Fig. S5). These results suggest that intestinal DGAT1 deficiency reduces the incorporation of dietary FA into neutral lipids and results in increased fecal FA and cholesterol excretion.

### 3.6 Pharmacological DGAT1 inhibition in HFD-fed mice increases fecal NLS via enhanced TICE

**Fig. 6:**
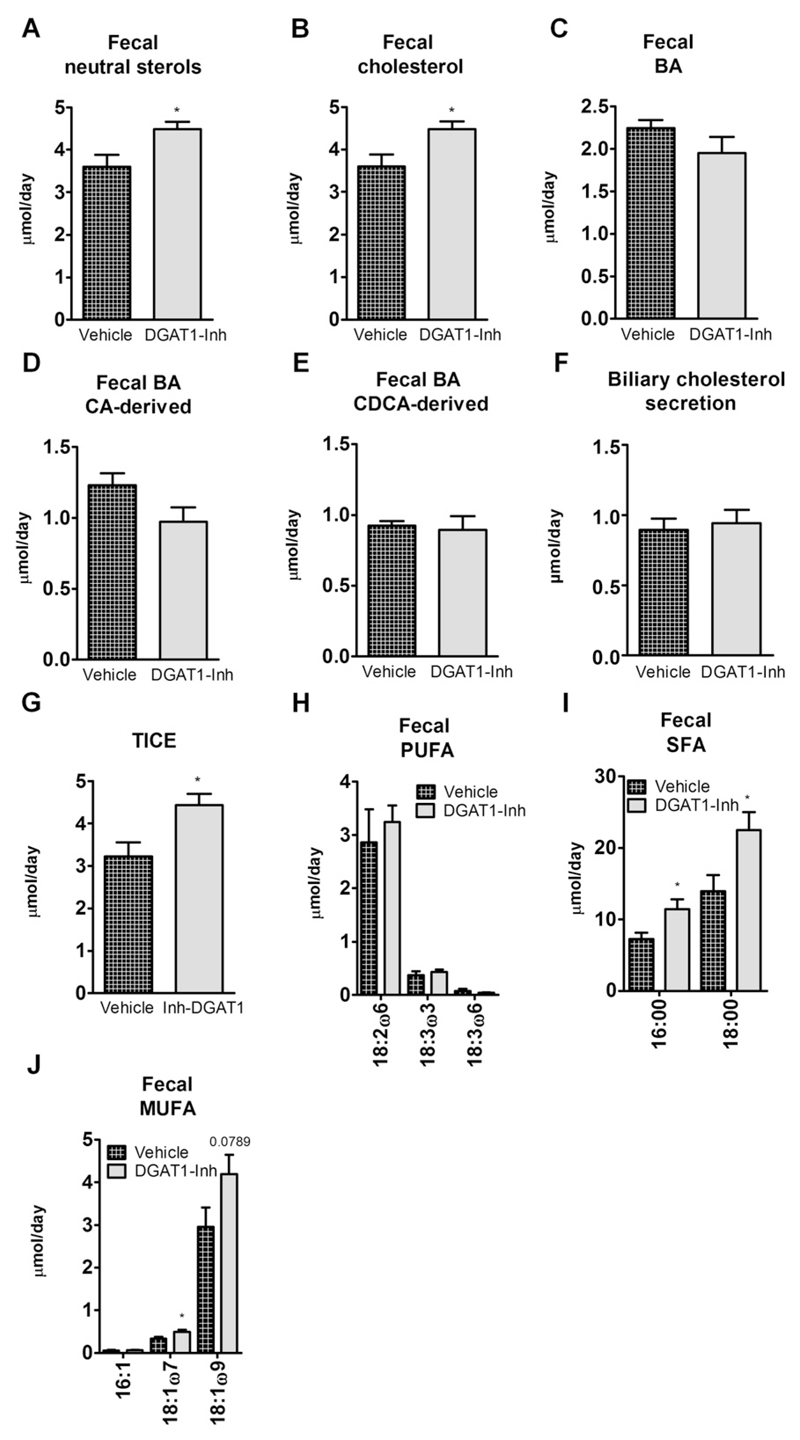
Pharmacological DGAT1 inhibition in HFD-fed mice increases fecal neutral sterol loss via enhanced trans-intestinal cholesterol excretion (TICE). Mice (*n* = 8) were fed HFD supplemented with either vehicle or DGAT1-Inh (5 mg/kg diet) for 4 weeks. Feces were collected for 72 h and neutral sterols, bile acids (BA), and fatty acids (FA) were measured by gas chromatography. (A) Total fecal neutral sterols, (B) cholesterol, (C) total bile acids (BA), (D) cholic acid (CA)-derived BA, (E) chenodeoxycholic acid (CDCA)-derived BA, (F) biliary cholesterol secretion, (G) TICE, (H) poly-unsaturated FA (PUFA), (I) saturated FA (SFA), and (J) mono-unsaturated FA (MUFA) are indicated. Values represent mean + SEM. *p < 0.05, Student’s *t*-test.

To determine whether chronic pharmacological DGAT1 inhibition phenocopies changes in cholesterol metabolism observed in I-DGAT1^−/−^ mice we measured fecal NSL and cholesterol flux in DGAT1-Inh mice. Comparable with the results from I-DGAT1^−/−^ mice, DGAT1-Inh mice exhibited increased fecal NSL (Fig. 6A) accompanied by elevated secretion of fecal cholesterol (Fig. 6B). Increased fecal NSL was independent of BA excretion as fecal total BA, CA-derived BA, and CDCA-derived BA concentrations were comparable between DGAT1-Inh and vehicle-treated mice (Fig. 6C-E). Moreover, biliary cholesterol secretion remained constant between the two groups (Fig. 6F) indicating the possibility of altered TICE, which indeed was increased by 1.4-fold in DGAT1-Inh mice (Fig. 6G). Although fecal excretion of PUFA was unaffected (Fig. 6H), fecal SFA and MUFA concentrations were significantly increased in DGAT1-Inh compared to vehicle-treated mice (Fig. 6I, J).

## 4 Discussion

In the present study, we show that intestinal DGAT1 deficiency is sufficient to alter whole-body cholesterol homeostasis in mice. DGAT1^−/−^ mice are resistant to diet-induced weight gain but the phenotype is reversed when DGAT1 is expressed only in the intestine [3,7]. It is well accepted that intestinal DGAT1 deficiency drives the reduction in high fat diet-induced weight gain in DGAT1^−/−^ mice. Our study provides evidence that intestinal DGAT1 deficiency alone is sufficient to prevent diet-induced weight gain and lowers cholesterol levels in HF/HCD-fed mice.

Reduction in postprandial TG concentrations upon DGAT1 inhibition in humans have been consistent with animal studies but their development as a therapeutic target for obesity, diabetes, and hyperlipidemia have been hampered due to unwanted gastro-intestinal side effects [24,25]. Pharmacological inhibitors of enzymes involved in chylomicron metabolism (such as ACAT2 and microsomal triglyceride transfer protein) in human trials have met with a similar fate [26,27]. Of note, we observed no diarrheal phenotype in I-DGAT1^−/−^, DGAT1^−/−^ or DGAT1-Inh mice since mice may be less dependent on DGAT1 function [16] than humans. Increased plasma GLP-1 levels due to DGAT1 inhibition in humans and animals models leads to delayed gastric emptying, which might lead to vomiting and diarrhea in humans [8,24].Thus, understanding and exploring the chylomicron neutral lipid balance could help in generating better tolerable inhibitors targeting the gut metabolism to treat hyperlipidemia and obesity.

We have shown that loss of DGAT1 on the ApoE-deficient background results in lower plasma cholesterol concentrations and reduced intestinal cholesterol absorption [15]. However, whether cholesterol concentrations and cholesterol metabolism per se are altered upon intestinal or systemic DGAT1 deficiency have not been explored. In the current study, we found that secretion of post-prandial [^3^H]triolein-and [^14^C]cholesterol-derived counts in chylomicrons were markedly reduced in I-DGAT1^−/−^ mice. Ables et al. have previously reported reduced chylomicron secretion with reduced postprandial TG and retinyl ester excursions in I-DGAT1^−/−^ mice [8]. DGAT1 possesses acyl-CoA:retinol acyltransferase activity [28], which could partly explain the reduction in chylomicron retinyl ester excursion due to intestinal DGAT1 deficiency. However, no acyl-CoA:cholesterol transferase activity has been reported for DGAT1. Our results suggest that enterocyte DGAT1 deficiency disturbs the intricate balance of dietary lipid esterification for chylomicron assembly, resulting in reduced TG, retinyl ester, and CE concentrations in chylomicrons.

We found increased fecal FA excretion in HF/HCD-fed I-DGAT1^−/−^ and HFD-fed DGAT1-Inh mice. In agreement with our observations, Denison et al. reported on increased FA concentrations in stools of human subjects treated with the same DGAT1-Inh used in our study [24]. These results suggest that DGAT1 inhibition leads to increased fecal FA excretion since TG re-esterification by DGAT1 and incorporation into chylomicrons is pivotal for channeling dietary FA for chylomicron assembly. Consistently, we observed significantly reduced body weight gain in DGAT1-Inh mice fed HFD for 4 weeks (data not shown). Thus, increased fecal FA excretion upon high-fat diet feeding might contribute to the lack of body weight gain observed in our mouse models. In contrast to our results, a previous study by Cases et al. reported on unchanged fecal fat in HFD-fed Dgat1^−/−^ mice [16]. The discrepancy between the two studies could partly be explained by differences in diet composition.

Reduced postprandial [^3^H]triolein- and [^14^C]cholesterol-derived counts assuming decreased TG and CE chylomicron concentrations in I-DGAT1^−/−^ mice resulted in smaller chylomicron size, similar to what we have observed in DGAT1^−/−^ mice. Studies in rats have shown that smaller chylomicron-sized particles are cleared faster from the circulation than large chylomicrons, possibly due to an increased surface area available for lipoprotein lipase to cleave TG within lipoproteins [29]. Smaller chylomicron particles and, thus, more efficient plasma clearance could account for reduced body weight gain and an improved metabolic profile in HF/HCD-fed I-DGAT1^−/−^ mice. Of note, reduction in postprandial chylomicron size is specific for intestinal DGAT1 deficiency because in the absence of intestinal acyl-CoA cholesterol transferase 2 (ACAT2), chylomicrons were depleted in CE, which were shown to be replaced mainly by TG without particle size alterations and unchanged fecal fat excretion [19]. We speculate that DGAT1 does not directly affect CE biosynthesis, but that the alterations in TG metabolism contribute to the altered cholesterol absorption observed in I-DGAT1^−/−^ and DGAT1^−/−^ mice.

Acute and fractional cholesterol absorption were considerably reduced in chow diet-fed I-DGAT1^−/−^, DGAT1^−/−^, and DGAT1-Inh mice. These results indicate that intestinal DGAT1 deficiency alone is sufficient to reduce cholesterol absorption to levels comparable to global DGAT1 deficiency or DGAT1 inhibition. Delayed gastric emptying is a well-known phenomenon in I-DGAT1^−/−^ and DGAT1^−/−^ mice as well as upon inhibition in mouse models and humans [8,14,24,30]. Treatment of I-DGAT1^−/−^ mice with the glucagon-like peptide-1 antagonist Exendin-9 corrected for delayed gastric emptying but the reduced postprandial TG and retinyl ester excursion was shown to persist [8]. In addition, a previous report suggests an inverse correlation between the rate of gastric emptying and cholesterol absorption [31].Thus, delayed gastric emptying might not be the sole factor responsible for reduced cholesterol absorption in I-DGAT1^−/−^ mice. To exclude the possibility that endocrine changes trigger reduced cholesterol absorption, we performed ex vivo enterocyte studies upon pharmacological DGAT1 inhibition. Reduced CE secretion but unaltered cholesterol uptake in DGAT1-Inh enterocytes suggests that DGAT1 inhibition reduces cholesterol absorption by decreasing CE secretion into chylomicrons. We speculate that the DGAT1 deficiency/inhibition-induced delay in gastric emptying could only be a contributing factor but not a decisive mechanism altering chylomicron secretion in murine models and human studies.

Decreased cholesterol absorption in I-DGAT1^−/−^ mice was reflected by reduced circulating plasma TC and CE concentrations in I-DGAT1^−/−^ mice, particularly in HDL and LDL fractions. This finding is in line with data of human volunteers where a significant reduction in plasma TC and LDL-C concentrations has been observed upon pharmacological DGAT1 inhibition (Turnbull AV, personal communication). In addition, we observed reduced body weight gain and circulating TC and CE concentrations in DGAT1^−/−^ LDLR^−/−^ compared to LDLR^−/−^ mice fed HF/HCD for 8 weeks. Together with our previous data on DGAT1^−/−^ ApoE^−/−^ mice [15], we conclude that DGAT1 deficiency/inhibition-mediated reduction in cholesterol concentration is independent of the ApoE-LDLR-mediated clearance of chylomicron remnants but is a direct result of reduced dietary cholesterol incorporation into chylomicrons.

We had speculated that altered NPC1L1 levels [32] could result in the observed decreased systemic cholesterol concentrations and increased fecal NSL in I-DGAT1^−/−^ mice. However, unchanged uptake of [^3^H]cholesterol in our ex vivo studies and unaltered intestinal NPC1L1 protein concentrations in I-DGAT1^−/−^ mice does not support this possibility. Our finding of unchanged hepatic Cyp7A1 mRNA expression in I-DGAT1^−/−^ mice together with unaltered biliary cholesterol secretion observed upon DGAT1 inhibition limits the possible involvement of hepato-biliary excretion pathways in regulating cholesterol metabolism in DGAT1 deficiency or inhibition. Reduced cholesterol absorption is known to increase the excretion of unabsorbed cholesterol in the feces [23]. TICE, the non-biliary route of direct secretion of cholesterol by the intestine, has been shown to account for ~30% of total fecal NSL excreted by wild-type mice [23]. Indeed, TICE was augmented by 1.4-fold in DGAT1-Inh mice. From these findings we conclude that changes in cholesterol metabolism in DGAT1-deficient and-inhibited mice are mainly a consequence of alterations in TICE.

In accordance with our results, a recent report has suggested that apolipoprotein B–containing lipoproteins might be the preferred cholesterol substrate for TICE. The basolateral lipoprotein receptors as well as intracellular trafficking pathways involved in TICE, however, have remained elusive [33]. Factors that regulate TICE include liver X receptor activation and peroxisome proliferator-activated receptor δ ligands [23,34]. We exclude these factors as being critically involved in explaining the I-DGAT1^−/−^ phenotype since mRNA and protein expression levels of several candidate genes were unaltered in duodena of I-DGAT1^−/−^ mice (Supplementary Fig. S5). We consider the smallersized chylomicrons in I-DGAT1^−/−^ mice being the source of increased fecal FA concentrations and NSL. To our knowledge, no study has so far linked smaller chylomicron particles in mice to changes in their exo-cytosis or in TICE. Future studies directed towards exploring chylomicrons as a substrate for TICE should shed more light to our hypothesis.

## 5 Conclusion

We conclude that deficiency and inhibition of DGAT1 affect cholesterol metabolism in mice due to reduced cholesterol absorption, smaller chylomicron size, and increased TICE. These effects are likely independent of cholesterol uptake by the intestine but mediated through altered dietary FA metabolism in the intestine. Our study shows that there exists an intricate balance between incorporation of TG and other neutral lipids into chylomicrons. Thus, a DGAT1-dependent decrease in chylomicron TG likely results in reduced incorporation of CE and RE into the chylomicron, which ultimately leads to smaller-sized chylomicron cargo. Understanding and exploring this chylomicron neutral lipid balance could help in generating better tolerable inhibitors targeting the gut metabolism to treat hyperlipidemia and obesity.

## Appendix A. Supplementary data

Supplementary data to this article can be found online at http://dx.doi.org/10.1016/j.bbalip.2016.06.014.

**Transparency document**

The Transparency document associated with this article can be found, in online version.

Supplementary Information

## Abbreviations

ACAT: acyl-CoA:cholesterol acyltransferase
ApoE: apolipoprotein E
DGAT: acyl-CoA:diacylglycerol acyltransferase
I-DGAT1: intestine-specific
Inh-DGAT1: DGAT1 inhibitor-treated
GLP-1: glucagon-like peptide 1
HF/HCD: high-fat/high-cholesterol diet
MTP: microsomal triglyceride transfer protein
NLS: neutral sterol loss
TICE: trans-intestinal cholesterol excretion
