# Supplementary material for: Novel role of a triglyceride-synthesizing enzyme: DGAT1 at the crossroad between triglyceride and cholesterol metabolism
Source: Biochim Biophys Acta. Author manuscript; Available in PMC 2016 Sep 1. (PMC4948681; doi:10.1016/j.bbalip.2016.06.014)
Supplement: Supplementary Information [file NIHMS69039-supplement-Supplementary_Information.pdf]

Supplementary Information

Novel role of a triglyceride-synthesizing enzyme: DGAT1 at the crossroad between triglyceride and cholesterol metabolism

by V. Sachdev *et al*.

Supplementary Methods

RNA isolation and quantitative real-time PCR

Total RNA from tissues was extracted using TriFast according to the manufacturer's protocol (Peqlab, Erlangen, Germany). Total RNA (2 µg) was reverse transcribed using the High Capacity cDNA Reverse Transcription Kit (Applied Biosystems, Carlsbad, CA). Quantitative real-time PCR was performed on a Roche LightCycler 480 (Roche Diagnostics, Palo Alto, CA) using the QuantiFastTM SYBR® Green PCR Kit (Qiagen, Valencia, CA). Samples were analyzed in duplicate and normalized to the expression of peptidylprolyl isomerase A *(*Ppia, also known as cyclophilin A). Expression profiles and associated statistical parameters were calculated using the public domain program Relative Expression Software Tool – REST 2008 (http://www.genequantification.com/download.html). Primer pairs used in this study are shown in Table S2.

Western blotting analysis

Mucosal scrapings were sonicated (Labsonic B. Braun, Melsungen, Germany) in RIPA buffer, and protein concentrations were determined by Bradford assay. Tissue lysates were separated by 10% SDS-PAGE and transferred onto a PVDF membrane. Non-specific binding sites were blocked by incubating the membrane with 5% non-fat dry milk in 1x TBS-T buffer (150 mM NaCl, 10 mM Tris, 0.1% Tween 20, pH 8) for 2 h at RT. Blots were incubated with rabbit polyclonal antibodies against NPC1L1 (1:200), CD36 (1:1,000), SR-B1 (1:1,000) and mouse monoclonal antibody against ABCG8 (1:1000; all purchased from Novus Biologicals, Littleton, CO). Monoclonal anti-mouse beta-actin (1:5,000) (Santa Cruz Biotechnology, Heidelberg, Germany) was used as loading control. HRP-conjugated goat

anti-rabbit (1:2,500) and rabbit anti-mouse antibodies (1:500) (Dako, Glostrup, Denmark) were visualized by enhanced chemiluminescence detection (Clarity^{TM} Western ECL substrate; Bio-Rad) using a ChemiDoc^{TM} MP imaging system (Bio-Rad, Hercules, CA).

Supplementary Figures

Figure S1, related to Figure 1

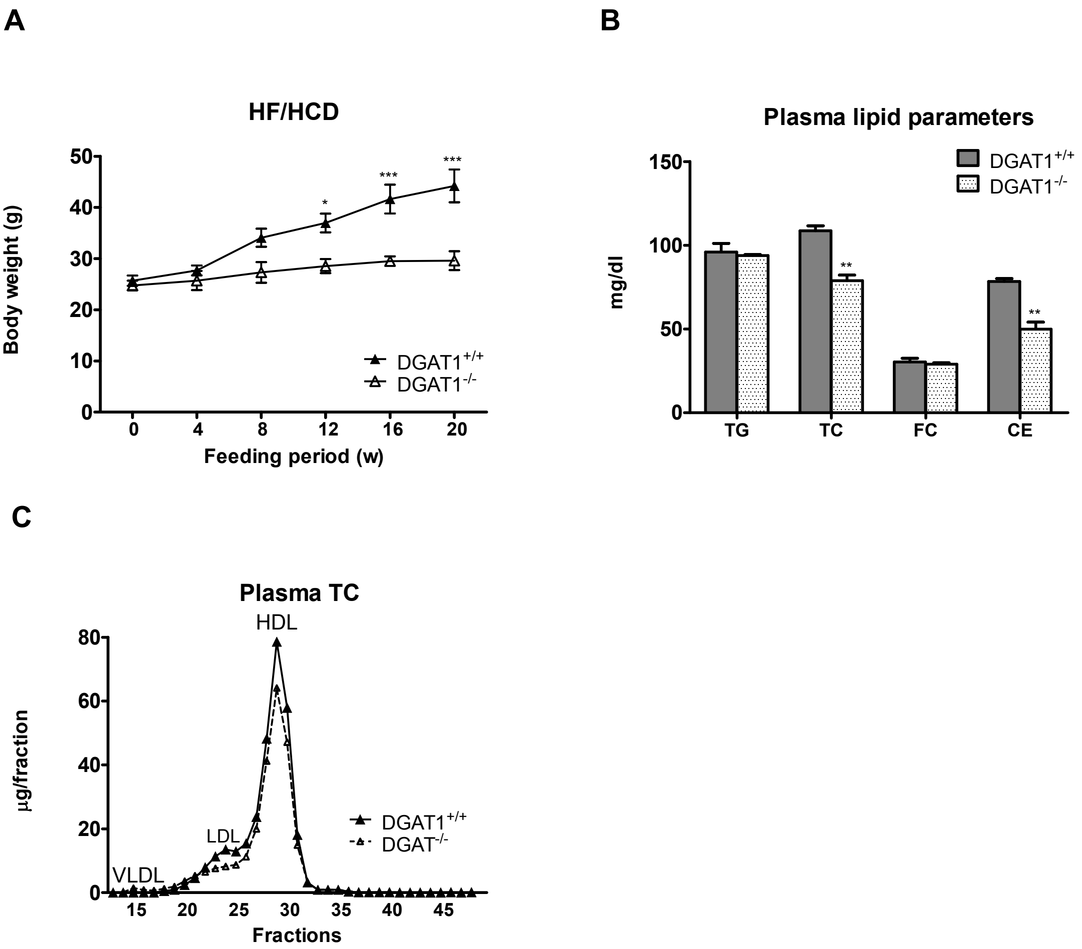

Figure S1: Whole-body DGAT1 deficiency reduces plasma cholesterol concentrations. DGAT1$^{+/+}$ and DGAT1$^{-/-}$ mice were fed HF/HCD for 20 weeks. **(A)** Body weights (n=3) and **(B)** plasma TG, TC, and FC concentrations were measured spectrophotometrically. CE concentrations were determined as subtraction of FC from TC (n=3). **(C)** Lipoprotein profile of TC after separation by fast performance liquid chromatography of pooled plasma (n = 3). Values represent mean ± SEM. A: 2-way ANOVA followed by Bonferroni post-tests; B: Student's t-test. $^{*}p<0.05$, $^{**}p<0.01$, $^{***}p<0.001$.

Figure S2, related to Figure 1

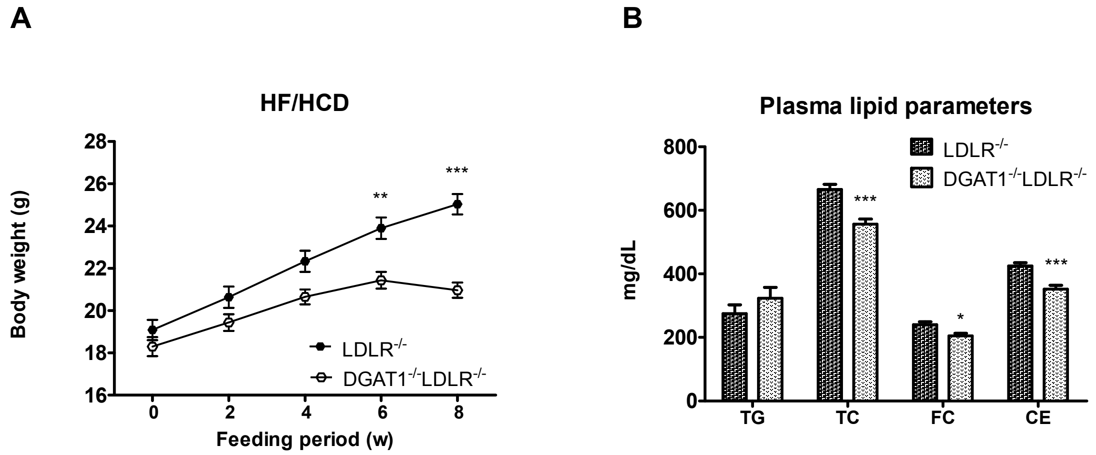

Figure S2: DGAT1 deficiency in LDLR$^{-/-}$ mice reduces plasma cholesterol concentrations.

LDLR$^{-/-}$ and DGAT1$^{-/-}$LDLR$^{-/-}$ mice were fed HF/HCD for 8 weeks. **(A)** Body weights and **(B)** plasma TG, TC, and FC concentrations were measured spectrophotometrically. CE concentrations were determined as subtraction of FC from TC (n=6). Values represent mean ± SEM. A: 2-way ANOVA followed by Bonferroni post-tests; B: Student's t-test. $^{*}p<0.05$, $^{***}p<0.001$.

Figure S3, related to Figure 4

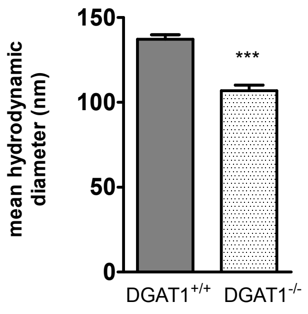

Figure S3: Whole-body DGAT1 deficiency in mice reduces chylomicron size.

DGAT1$^{+/+}$ and DGAT1$^{-/-}$ mice (n=4-5) fed HF/HCD for 8 weeks were intraperitoneally injected with poloxamer-407 (1 g/kg body weight, in PBS) and gavaged with 200 µl corn oil. Plasma was collected 90 min post-gavage and postprandial plasma chylomicron size was measured by Zetasizer nano.

Figure S4, related to Figure 5

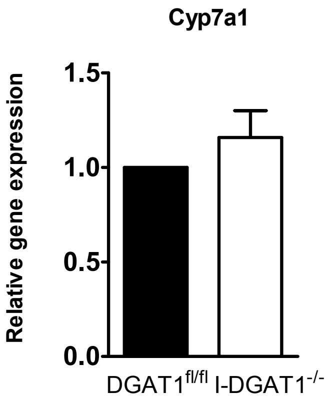

Figure S4: Unchanged hepatic Cyp7a1 mRNA levels in I-DGAT1$^{-/-}$ mice.

DGAT1$^{fl/fl}$ and I-DGAT1$^{-/-}$ mice were fed HF/HCD diet for 20 weeks. Total RNA was isolated from liver and subjected to real time PCR analysis. mRNA expression of Cyp7a1 was analyzed in duplicate and normalized to the expression of Ppia as reference gene. Data represent mean values + SD (n = 3).

Figure S5, related to Figure 5

A

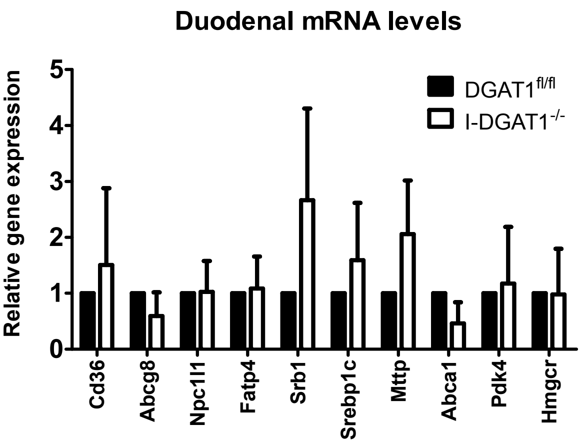

B

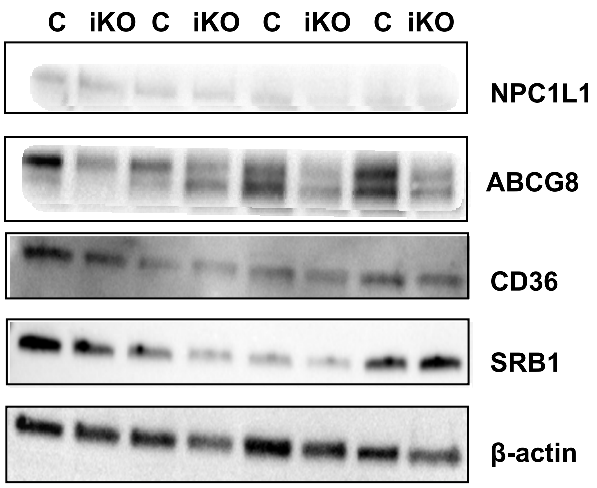

C

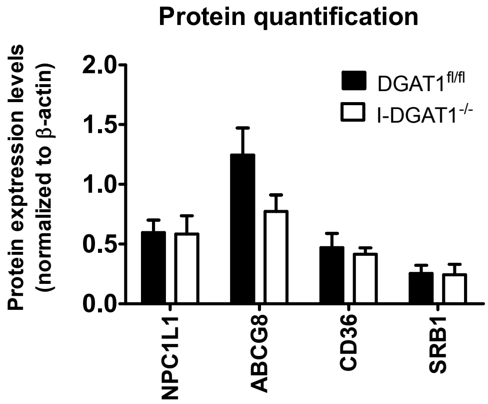

Figure S5: Unchanged mRNA and protein expression in duodena of I-DGAT1$^{-/-}$ mice. **(A)** Duodenal mRNA expression after 4 h fasting of mice fed HF/HCD for 20 weeks. Data represent mean values ± SD (n = 3). **(B)** Duodenal lysates of HF/HCD-fed DGAT1$^{fl/fl}$ mice (denoted by C) and I-DGAT1$^{-/-}$ (denoted by iKO) were separated by SDS-PAGE. NPC1L1, ABCG8, CD36, SR-B1 and β-actin protein expression was analyzed by western blotting. The expression of β-actin served as loading control. (C) Quantification of protein expression.

Supplementary Tables

Table S1: Fatty acid composition of experimental diets used in the study (% of total fatty acids)

Fatty acid	HF/HCD	HFD
C 10:0	-	0.03
C 12:0	0.09	0.07
C 14:0	3.32	1.1
C 15:0	-	0.07
C 16:0	26	19.67
C 16:1	2.51	1.33
C 17:0	1.22	0.35
C 18:0	17.09	10.56
C 18:1	39.12	34.02
C 18:2	8.22	28.72
C 18:3	2.06	2.04
C 20:0	0.12	0.15
C 20:1	0.03	0.58
C 20:2	-	0.78
C 20:3	-	0.11
C 20:4	0.22	0.27
C 22:5	-	0.07
SFA	47.84	32
MUFA	41.66	35.93
PUFA	10.50	31.99

Table S2: Primer sequences used for real time PCR

Gene	Forward primer	Reverse primer
Cd36	GCAGGTCTATCTACGCTGTG	GGTTGTCTGGATTCTGGAGG
Abcg8	CTGTGGAATGGGACTGTACTTC	GTTGGACTGACCACTGTAGGT
Npc1l1	GCAAGGTGATCAGGAGGTTGA	ATCCTCATCCTGGGCTTTGC
Fatp4	ACTGTTCTCCAAGCTAGTGCT	GATGAAGACCCGGATGAAACG
Srb1	GAGCACGTTCTACACGCAG	GGTCTGACCAAGCTATTCAGGTT
Srebp1c	ATCGGCGCGGAAGCTGTCGGGGT AGCGTC	ACTGTCTTGGTTGTTGATGAGCTGG AGCAT
Mttp	CACACAACTGGCCTCTCATTAAAT	TGCCCCCATCAAGAAACACT
Abca1	CTCTTCATGACTCTAGCCTGGA	ACACAGACAGGAAGACGAACAC
Pdk4	CCGCTGTCCATGAAGCA	GCAGAAAAGCAAAGGACGTT
Hmgcr	TAGCTCGTGGAATGGCAATC	CTCTAGGACCAGCGACACAC
Cyp7a1	AGACCTCCGGGCCTTCCT	ATCACTCGGTAGCAGAAGGCAT
Ppia	CCATCCAGCCATTCAGTCTT	TTCCAGGATTCATGTGCCAG
